# Validity and Reliability of Rubrics for Assessment of Preclinical Orthodontic Practices in Dental Students

**DOI:** 10.3390/dj14080516

**Published:** 2026-08-13

**Authors:** J. M. Aguado-Gil, S. Arena-Echeverry, A. Suárez-García, M. Gómez-Sánchez, G. González-Cuevas

**Affiliations:** 1 Department of Preclinical Dentistry II, Faculty of Biomedical and Health Sciences, Universidad Europea de Madrid, C/Tajo s/n, Villaviciosa de Odón, 28670 Madrid, Spain; stefania.arena@universidadeuropea.es (S.A.-E.); ana.suarez@universidadeuropea.es (A.S.-G.); 2 Faculty of Dentistry, Alfonso X El Sabio University, Avenida de la Universidad 1, 28691 Villanueva de la Cañada, Spain; margaritagomez@uax.es; 3 Social and Behavioral Sciences Division, Central Arizona College, Coolidge, AZ 85128, USA; gustavo.gonzalezcuevas@centralaz.edu; 4 Social and Behavioral Sciences Division, Boise State University, Meridian, ID 83725, USA

**Keywords:** rubrics, dental education, dentistry degree, preclinical practice, evaluation tools, psychometry

## Abstract

**Background:** Objective assessment of preclinical dental practices remains challenging, as it requires consistency, transparency, and standardization in the evaluation of practical competencies. Rubrics have been proposed as useful tools for improving the quality of assessment; however, evidence regarding their psychometric properties in preclinical orthodontic education remains limited. **Aim:** This study aimed to analyze the reliability, internal structure, and academic usefulness of assessment rubrics applied to preclinical orthodontic practice in undergraduate dental education. **Methods:** A cross-sectional psychometric retrospective study was conducted with 175 third-year dentistry students enrolled in Orthodontics I at the European University of Madrid. Two analytical rubrics designed to assess wire-bending and cephalometry practices were evaluated. Reliability was assessed using Cronbach’s alpha, construct validity using exploratory factor analysis, and associations with academic performance by correlation analyses. **Results:** The wire-bending rubric showed moderate internal consistency (α = 0.61), whereas the cephalometry rubric demonstrated adequate reliability (α = 0.71). Exploratory factor analysis yielded evidence consistent with a one-dimensional structure for both instruments. Cephalometry performance was positively associated with theoretical examination scores and seminar performance, whereas no significant associations were observed for wire-bending performance. Students reported generally positive perceptions of rubric use, although lower post-assessment ratings were observed in the wire-bending activity. **Conclusions:** These findings provide preliminary evidence supporting the reliability and educational usefulness of rubric-based assessment in preclinical orthodontic education. These instruments may contribute to greater transparency and consistency in student assessment. Further studies involving multiple evaluators, multicenter samples, longitudinal designs, and psychometric approaches specifically developed for ordinal data are required.

## 1. Introduction

Assessment plays a central role in contemporary university education, especially in degrees with a high practical component, such as the bachelor’s degree in Dentistry. In this context, assessment not only fulfils an accrediting role but also acts as a structural element of the learning process, supporting the progressive acquisition of practical competences. Therefore, the development of instruments that unify criteria, increase objectivity and provide effective feedback has become a priority in health professions education [1,2,3].

The concept of assessment has evolved from grading-focused approaches to continuous, systematic and formative models, based on the collection and interpretation of evidence to improve learning and teaching practice. In this framework, informed decision-making acquires a key role, overcoming the reductionist vision of evaluation as mere assignment of a score [4,5].

In dentistry, this need is especially relevant in preclinical practices, where students must demonstrate not only theoretical comprehension but also psychomotor skills, technical precision and the ability to apply standardized criteria before entering clinical practice. The evaluation of this type of activity poses specific difficulties, since it involves multidimensional tasks with a potential component of subjectivity on the part of the evaluator [6,7,8,9].

In response to these limitations, rubrics have gained attention in health and dental education because they explicitly define assessment criteria, performance levels, and learning expectations. Their use has been associated with greater transparency of the evaluation process, improved feedback and greater coherence between evaluators. However, in the preclinical dental field, the literature has focused mainly on their perceived usefulness or their application in specific contexts, and there are still few studies that systematically examine their psychometric properties [10,11,12,13].

This limitation is relevant because the incorporation of rubrics should be based not only on their acceptance but also on evidence supporting score interpretation. Analyzing their reliability, internal structure, and relationships with academic variables makes it possible to determine whether these instruments generate measures that are coherent with the construct being assessed [14,15,16].

In preclinical orthodontic practice, wire bending and cephalometry are two contexts that are particularly suitable for this type of analysis. Both activities are part of students’ fundamental training, require technical precision and the application of observable criteria. They involve distinct competencies, as wire bending is mainly related to manual dexterity, whereas cephalometry integrates analytical reasoning, anatomical localization, and measurement skills [17,18,19,20].

Examining whether the scores obtained using these instruments are associated with other indicators of academic performance may help determine their usefulness within a competency-based assessment approach [21].

The objective of this study was to analyze the psychometric properties and academic usefulness of two evaluation rubrics applied to preclinical orthodontic practice in the Dentistry Degree program at Universidad Europea de Madrid. Specifically, students’ performance, instrument reliability and construct validity were evaluated, as well as the relationship between rubric scores and academic performance variables [22,23].

## 2. Materials and Methods

### 2.1. Design

The research was designed as an observational, correlational, quantitative, cross-sectional retrospective study with an instrumental component aimed at analyzing the psychometric properties of two assessment rubrics used in preclinical orthodontic practice. The study was conducted in the bachelor’s degree in Dentistry program at Universidad Europea de Madrid. The study was approved by the Research Commission of the Research Unit of the Universidad Europea, on 19 June 2025, RC Code 2026-939.

### 2.2. Participants

The sample comprised 175 third-year students enrolled in Orthodontics I during the academic years 2017–2018 and 2018–2019.

Only students evaluated by the same teacher were included to reduce variability associated with differences in the application of the evaluation criteria.

Groups taught in English were excluded due to possible difficulties understanding the perception surveys.

### 2.3. Development and Validation of Rubrics

The design and development of the rubrics were structured in several phases. In an initial stage, a Community of Practice was formed by 30 professors from the bachelor’s degree in Dentistry, with the aim of agreeing on common evaluation criteria for preclinical practices. This collaborative process made it possible to identify relevant transversal competencies (responsibility, punctuality, attitude, appropriate use of material and respect for the environment), and to establish general guidelines aligned with the White Paper on Dentistry and the principles of the EHEA.

Based on this consensus, an initial regulation was drawn up that defined serious faults, weightings, rating scales and a limited number of evaluation criteria.

Subsequently, specific rubrics were developed for preclinical activities in wire-bending and cephalometry in Orthodontics I. These pilot rubrics were implemented during the 2015–2016 academic year.

In a later phase, the rubrics were subjected to external review by orthodontic experts from outside the institution, who evaluated the adequacy of the criteria, the clarity of the descriptors and the weighting of the items using a structured questionnaire.

The expert review process was qualitative and was intended to obtain feedback on the clarity, relevance, and applicability of the assessment criteria. Formal measures of expert agreement or quantitative content validity indices were not collected.

**Figure 1:**
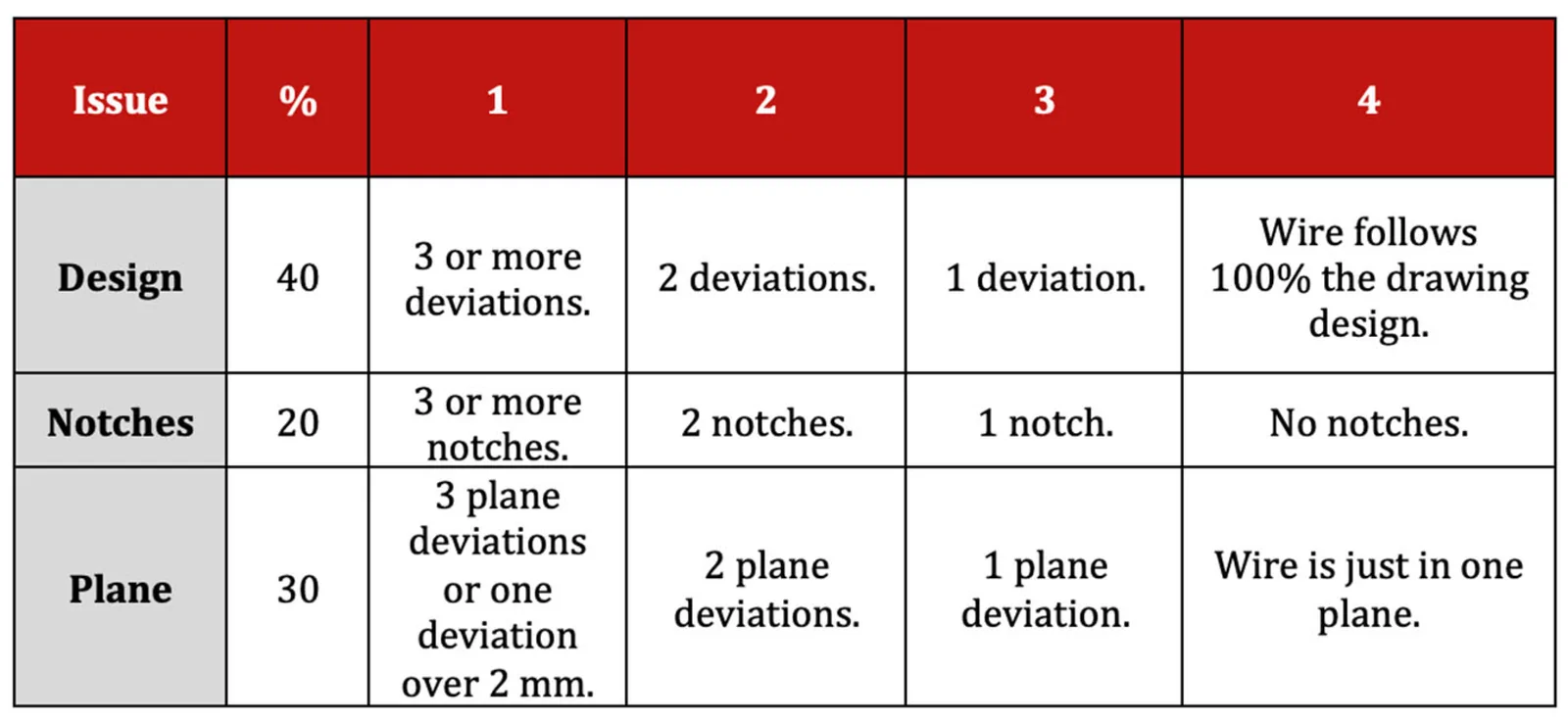
Wire-bending rubric.

The experts’ observations were used to make minor adjustments to the weighting of some criteria and to the wording of selected descriptors to improve their clarity and applicability. The revised and agreed-upon versions of the rubrics were used in this study (Figure 1).

### 2.4. Assessed Preclinical Practices

The practical activities analyzed corresponded to two thematic blocks: wire bending and cephalometry. Each block comprised three training sessions and one final assessment session, following a progressive model that included prior instruction and feedback.

Wire bending was evaluated based on three main criteria (design, notches, and plane), while cephalometry included five dimensions (tracing, location of points, lines, and planes, cephalometric measurements, and cleanliness).

These activities were selected because they represent distinct competencies within preclinical orthodontic education. Wire bending mainly assesses psychomotor skills, whereas cephalometry involves analytical reasoning, anatomical identification, and measurement skills. Both activities are structured and based on observable performance criteria, making them suitable for rubric-based assessment and psychometric evaluation.

All criteria were assessed using four-level ordinal scales [1,2,3,4], in accordance with the original rubric design. Given the ordinal nature of these scales, scores were interpreted as indicators of the performance level achieved for each criterion [16,24].

Final scores were calculated by summing the weighted percentages assigned to the established criteria. A score of 2 represented the minimum level required to pass the criterion (pass), whereas a score of 1 indicated insufficient performance (fail), a score of 3 indicated very good performance, and a score of 4 indicated full achievement of the criteria (outstanding).

### 2.5. Academic Variables and Student Perception

Additional academic variables were collected, including the theoretical examination grade, the cephalometry seminar grade and the final practical course grade. Satisfaction surveys were also administered before and after each practical block using five-point Likert-type scales to assess students’ perceptions of the clarity, usefulness and appropriateness of the rubrics.

The surveys were anonymous and voluntary and were administered on paper during the practical course. Students completed and returned them immediately.

For incomplete records, analyses were conducted using the available cases for each variable.

### 2.6. Statistical Analysis

The statistical analysis was performed using IBM SPSS Statistics 30.0. Analyses were carried out using descriptive statistics, mean comparison tests (Student’s *t*-test and repeated measures ANOVA), reliability analysis using Cronbach’s alpha coefficient [25] and construct validity assessment through exploratory factor analysis, after checking sample adequacy (KMO) and Bartlett’s test of sphericity.

The associations between rubric scores and other variables were analyzed using Pearson correlations and partial correlations.

Statistical significance was set at *p* < 0.05.

## 3. Results

The initial sample comprised 175 students, with a balanced sex distribution and a mean age of 23.14 years. For the analyses of the pre- and post-assessment perception surveys, 156 students were included because some questionnaires were incomplete.

### 3.1. Academic Performance in Preclinical Internships

In wire bending, 82.86% of the students passed, with an average grade of 6.80/10 (SD = 1.86).

Grades were compared with those recorded in preceding academic cohorts of the same course, whose mean was 7.48/10 (SD = 1.26). Student’s *t*-test for independent samples showed statistically significant differences between groups, *t*(355) = 4.04, *p* < 0.01, *d* = 0.43, with higher mean scores in the previous cohorts.

For the different criteria evaluated (design: M = 2.54, SD = 1.86; notches: M = 2.83, SD = 0.85; plane: M = 2.73, SD = 0.85), a repeated-measures ANOVA was applied. The effect of the criterion was significant, *F*(2, 348) = 7.81, *p* = 0.01, *η*^2^*p* = 0.04.

Post-hoc comparisons with Bonferroni correction showed that scores were significantly lower for the design criterion compared with notches (*p* < 0.01) and plane (*p* < 0.05). No significant differences were observed between notches and plane (*p* = 0.53). There were statistically significant differences observed according to sex.

In cephalometry practice, 78.86% of the students passed, with an average grade of 6.57/10 (SD = 2.13). These scores were compared with those from previous cohorts of the same course (M = 7.19, SD = 2.17): Student’s *t*-test for independent samples showed statistically significant differences, *t*(355) = 2.74, *p* < 0.01, *d* = 0.29, with higher mean scores in the previous cohorts.

The analysis of the different criteria evaluated in cephalometric practice showed the following scores: tracing: M = 2.41, SD = 0.92; points: M = 3.11, SD = 0.94; lines and planes: M = 2.86, SD = 1.07; measurements: M = 2.37, SD = 1.05; cleanliness: M = 2.82, SD = 0.94. Repeated-measures ANOVA showed a significant effect of criterion, *F*(4, 696) = 26.90, *p* = 0.01, *η*^2^*p* = 0.13. Post hoc comparisons with Bonferroni correction showed that the scores were significantly higher for points than for the remaining criteria (*p* < 0.05) and lower for tracing and measurements than for the remaining criteria (*p* < 0.01). No significant differences were found between tracing and measurements (*p* = 1).

Significant differences were only observed in the cleanliness criterion, with women obtaining higher scores. No significant differences were identified in overall performance between the two practices.

The average grade of the theoretical exam was 5.84 (SD = 2.05), while the overall average grade of the course was 7.00 (SD = 1.31), with no statistically significant differences according to sex.

### 3.2. Students’ Perception of the Use of Rubrics

The surveys showed a high initial assessment of the rubrics in both practices.

For wire bending, a significant decrease in ratings was observed after the evaluation, especially in the items related to theoretical knowledge and expectations, while manual skills remained the most valued aspect (Table 1).

In cephalometry practice, global perception remained stable after the assessment, with only a slight decrease in the performance item (Table 2).

Item-by-item repeated-measures ANOVA revealed significant differences in the pre-assessment survey (*F*(4, 620) = 11.61, *p* = 0.01, *η*^2^*p* = 0.07) and the post-assessment survey (*F*(4, 620) = 8.17, *p* = 0.01, *η*^2^*p* = 0.05). The highest mean scores were observed for objectives in the pre-assessment survey and expectations in the post-assessment survey. The lowest score was observed for manual skills at both time points.

In the two practical blocks, grade was positively associated with subsequent assessment of the evaluation system.

### 3.3. Reliability and Psychometric Validity of the Instruments

The wire-bending rubric showed moderate reliability (α = 0.61), while the cephalometry rubric reached an adequate level of internal consistency (α = 0.71). Satisfaction surveys showed Cronbach’s alpha values above 0.70 in all versions (Table 3).

For the wire-bending rubric, item performance analysis indicated that no item removal was recommended, as the total scale alpha decreased when any item was removed and all item–total correlations were above the 0.30 cut-off point (Table 4). Similar results were observed for the cephalometry rubric, although the cleanliness item showed a slightly lower contribution to the overall scale (Table 5).

The exploratory factor analysis supported the one-dimensionality of the rubrics and surveys, with adequate KMO index and statistically significant Bartlett tests of sphericity.

For the wire bending rubric, the Kaiser–Meyer–Olkin index (KMO = 0.64) and Bartlett test, χ^2^(3) = 56.21, *p* < 0.01, indicated that the data were adequate for factor analysis. A single factor with an eigenvalue greater than 1 was extracted, explaining 56.33% of the total variance. Factor loadings exceeded 0.5 (Table 6).

In the cephalometry rubric, the KMO index was 0.74 and Bartlett’s test was significant (χ^2^ (10) = 164.78, *p* < 0.001). A single factor explained 47.07% of the total variance, with factor loadings above 0.5 for all items (Table 7).

In the satisfaction surveys for wire bending, the pre- and post-assessment KMO indices were 0.79, Bartlett’s tests were significant, and a single factor explained 53.28% of the total variance.

Similar results were observed for cephalometry, with KMO indices of 0.77 in the pre-assessment and 0.76 in the post-assessment survey and significant Bartlett’s tests.

### 3.4. Relationship with Academic Variables

**Figure 2:**
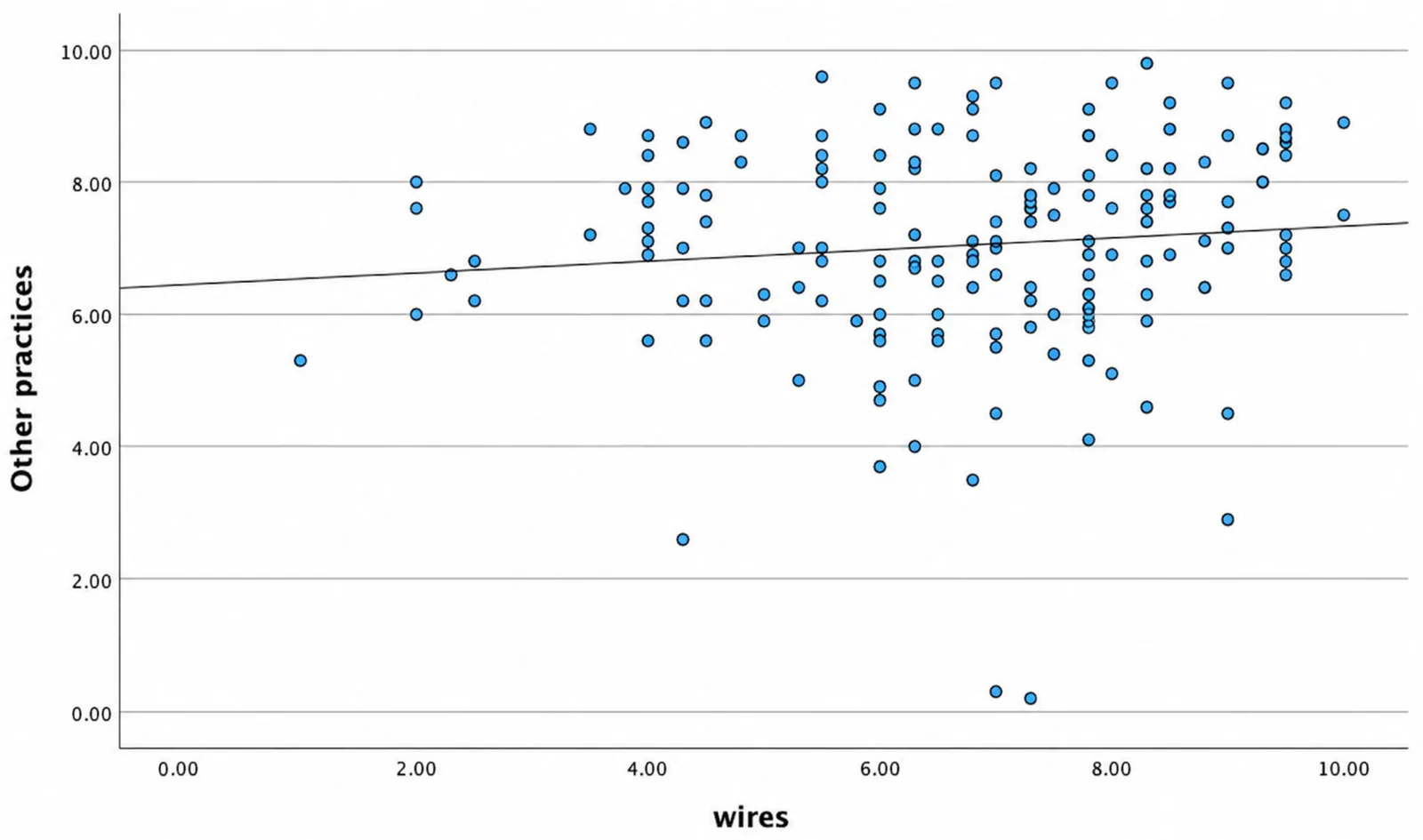
Scatterplot of the relationship between wire practice and other practices.

Performance in wire bending practice was not significantly associated with performance in other practices (*r*(175) = 0.11, *p* = 0.17). Line: average of grade (Figure 2).

**Figure 3:**
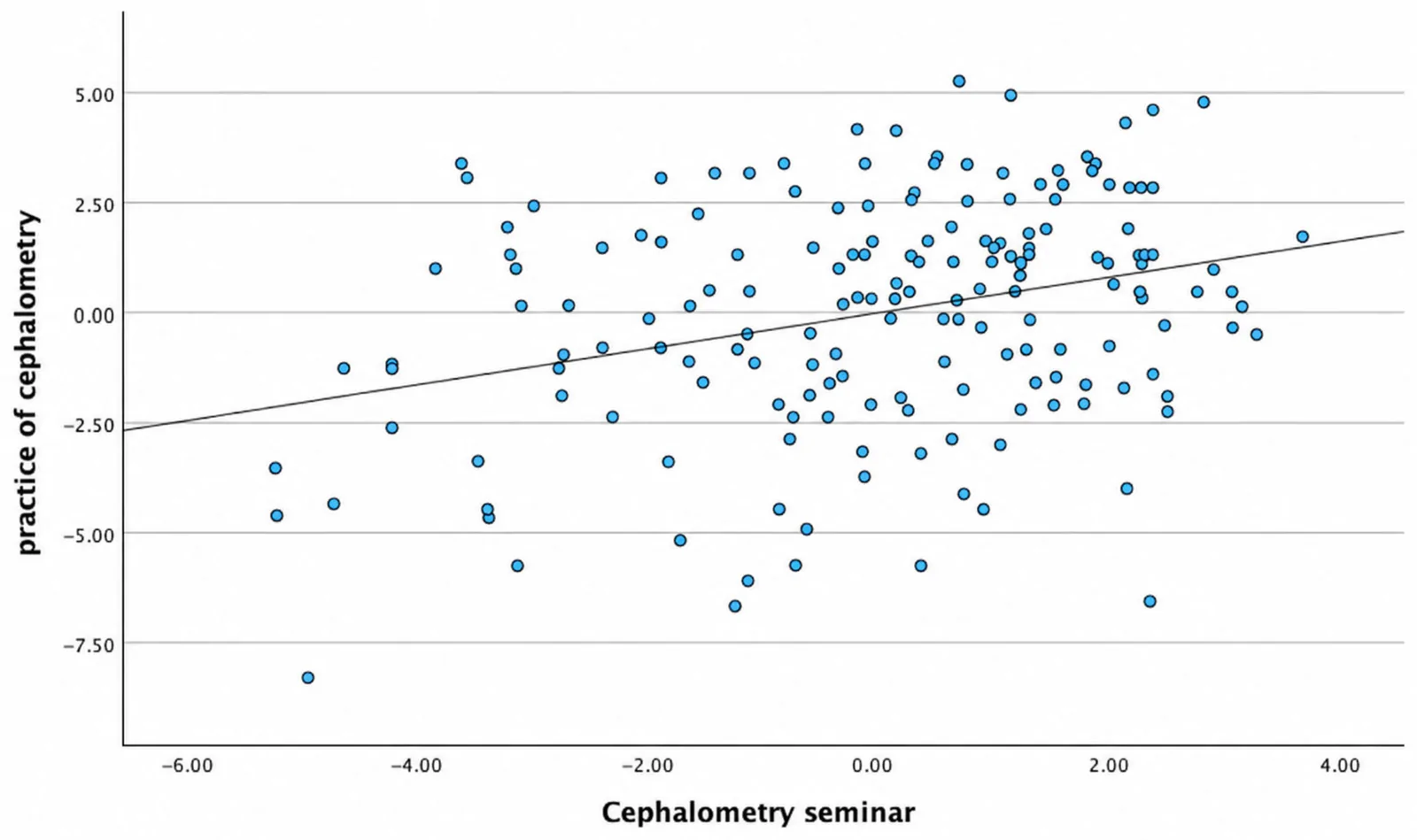
Scatterplot of the relationship between cephalometry seminar performance and cephalometry practice performance.

Conversely, performance in the cephalometry seminar showed a moderate positive association with cephalometry practice performance (*r*(175) = 0.36, *p* < 0.01) (Figure 3).

Cephalometry practice performance was also positively associated with theoretical performance, even after controlling for seminar performance (*partial* *r*(172) = 0.31, *p* < 0.01).

## 4. Discussion

The evaluation of preclinical practices in dentistry poses a methodological challenge, since it involves assessing cognitive, psychomotor and attitudinal dimensions in highly applied learning contexts.

This complexity has been pointed out in the literature, especially due to the difficulties in guaranteeing objectivity, coherence and standardization in the assessment of student performance in clinical and preclinical settings. Rubrics have been proposed as instruments capable of making evaluation criteria explicit, structuring evaluative judgment, and reinforcing the formative function of the evaluation process [1,9,23,26].

Previous studies evaluating preclinical dental performance using analytic rubrics have mainly focused on operative dentistry, prosthodontics, endodontics, and crown preparation procedures. Habib [16] developed a rubric system for crown preparation assessment, while Al Moaleem et al. [27] evaluated analytic rubrics in anterior ceramic crown preparations. More recently, Jabali et al. [28] applied a rubric-based assessment system to preclinical root canal treatment, and Tsai et al. [29] investigated rubric-guided self-assessment during preclinical endodontic training. These structured rubric systems may improve transparency, standardization, and consistency in student assessment. However, evidence regarding the psychometric evaluation of rubrics specifically designed for preclinical orthodontic activities remains limited.

The present study investigated two rubrics applied to preclinical orthodontic practice from three complementary perspectives: academic performance, student perception and initial evidence of reliability and internal structure.

The design of the study, based on a Community of Practice formed by teachers of the bachelor’s degree in Dentistry, reflects the importance of teaching consensus in the definition of evaluation criteria. Although the initial review of the rubrics was carried out internally, the subsequent external review by orthodontic experts provided feedback on the clarity of the descriptors, the relevance of the selected criteria, and the weighting assigned to the different assessment dimensions, in line with previous recommendations on the design and refinement of assessment instruments. However, evidence supporting content validity should be interpreted with caution, as the expert review process was qualitative, and no formal measures of expert agreement or quantitative content validity indices were collected. Consequently, this stage improved the clarity and applicability of the instruments rather than providing formal evidence of content validity. Future studies should incorporate structured approaches, such as content validity indices or formal expert consensus procedures, to strengthen the evidence supporting these instruments [30,31,32,33,34].

The sample size allowed a preliminary analysis of the internal consistency and internal structure of the rubrics with a reasonable degree of stability.

The decision to exclude students from international groups aimed to minimize possible biases related to difficulties understanding perception surveys. However, this decision may have reduced the generalizability of the findings and introduced selection bias, since international students may differ from local students in educational background, previous training experiences, cultural factors, or perceptions of assessment systems. Future studies should include multilingual and international cohorts to determine whether the psychometric properties remain stable across different educational contexts.

Similarly, the inclusion of a single evaluator favored homogeneity in the application of the criteria and reduced variability between raters. However, this decision prevented the evaluation of inter-rater reliability. Consequently, it remains unclear whether the results would be reproducible across evaluators. Future studies should incorporate multiple calibrated assessors to determine he robustness of the instruments [29,30,35,36,37,38,39,40].

The results showed lower average grades than those recorded in previous academic years. Although this difference could be compatible with a stricter or more explicit application of the evaluation criteria, the study design does not allow causal attribution to the use of rubrics, since other potentially influential factors, such as teacher changes, cohort characteristics, or differences in the academic context, were not controlled. Therefore, this comparison should be interpreted with caution and understood as a contextual element rather than a demonstration of the effect of the evaluative intervention [41].

Regarding differences by sex, the absence of significant differences in most indicators is consistent with the literature, where no consistent statistically significant differences have been reported [42,43]. The only sex-related difference concerned the cleanliness criterion in the cephalometry exercise, where women achieved slightly higher scores. Given the isolated nature of this finding and the absence of additional explanatory variables, no causal interpretation can be established. Further research is required to determine whether this observation can be reproduced in other cohorts.

The students’ perception of rubrics was initially high in both practices. However, after the evaluation, a decrease was observed in some indicators, especially in wire bending. This pattern has been previously described [44,45] and may be related to several factors, including a more realistic understanding of the demands of the task after practical experience, a greater critical capacity among students, and the possible influence of the grade on perception of the evaluation system.

The fact that the subsequent assessment was positively associated with the grade suggests that the perception of the instrument does not depend only on its formal characteristics but also on the academic result. Despite this variability, the surveys showed adequate internal consistency, which supports their usefulness for exploring student perceptions in similar contexts [12,46,47,48].

From the psychometric point of view, the analyzed rubrics showed favorable initial evidence, although of unequal magnitude. The cephalometry rubric reached an adequate level of internal consistency (α = 0.71), while the wire bending rubric presented a more modest value (α = 0.61). Therefore, the reliability evidence provided by the latter should be considered preliminary.

Nevertheless, this finding may be partially explained by the limited number of items included in the rubric, three criteria, and by the nature of the task assessed, which involves different psychomotor skills and may therefore generate greater variability in student performance. Previous studies have recommended using a larger number of items [31,49,50,51].

Regarding the internal structure, the exploratory factor analysis showed results compatible with a one-dimensional organization of the rubrics and satisfaction surveys. However, this interpretation should be formulated with caution, especially for the wire bending rubric, given its three-item structure.

A three-item instrument provides limited information for establishing dimensionality and should not be considered robust evidence of a latent construct. The results should be interpreted as preliminary evidence of internal coherence that may justify the use of a global score within this educational context.

These findings are consistent with previous studies showing that rubrics can exhibit acceptable psychometric functioning when their criteria are clearly defined and aligned with the performance assessed [33,52].

However, because the rubric scores were derived from ordinal rating scales, the use of Cronbach’s alpha and Pearson correlations should be interpreted with caution. Although these methods remain widely used in educational research, future studies should incorporate psychometric approaches for ordinal data, such as ordinal alpha, omega coefficients, polychoric correlations, and factor analyses based on polychoric matrices [10,53].

The results suggest that not all practices provide the same type of information on student performance. Performance in wire bending was not significantly associated with other practices, while the cephalometry seminar was positively related to performance in the corresponding practice, and this, in turn, was associated with theoretical performance even after controlling for the seminar. Rather than demonstrating a strong predictive capacity of the rubrics, these results point to an academically significant relationship between certain training activities and performance in tasks that integrate practical and cognitive components. These findings are consistent with previous studies that question the use of isolated tests of manual dexterity as predictors of future performance but recognize their usefulness as partial indicators within a broader training process. This interpretation should also consider that students may also score themselves higher than teaching staff even when provided with assessment rubrics [54,55,56,57].

The present work has several limitations that must be considered when interpreting the results. These include the single-center design, which limits the generalizability of the findings to other institutions and educational contexts; the exclusion of students from English-taught groups, which may have introduced selection bias and limits the applicability of the results to more diverse or international student populations; and the use of a single assessor, which prevented the assessment of inter-rater reliability and does not allow conclusions to be drawn regarding the reproducibility of scores across evaluators. In addition, the cross-sectional design precluded assessment of the temporal stability of rubric scores and does not permit causal inferences regarding the effect of rubric use on academic performance.

Likewise, the ordinal nature of the scales, the small number of items in the wire bending rubric and the absence of additional sources of validity evidence make it advisable to interpret the psychometric results as preliminary evidence representing relevant psychometric limitations. In particular, the three-item structure of the wire-bending rubric may have reduced the stability of the internal consistency and factor-analytic estimates, while the use of Cronbach’s alpha, Pearson correlations and factor analysis based on conventional correlations may not fully account for the ordinal nature of the data. Furthermore, the qualitative expert review did not include formal indices of content validity, and the study did not assess other relevant sources of validity evidence. Therefore, the findings should be interpreted as preliminary evidence of reliability and internal structure rather than as a comprehensive validation of the instruments.

Overall, the results support the use of rubrics as useful instruments to structure preclinical evaluation in dental education and provide greater transparency in the evaluation process. However, their consolidation as psychometrically robust tools will require studies with multicenter designs, multiple evaluators and more advanced analytical strategies.

## 5. Conclusions

The cephalometry rubric demonstrated adequate internal consistency, whereas the wire-bending rubric showed moderate reliability. Both instruments showed evidence compatible with a unidimensional structure while positive associations with selected academic performance indicators were observed particularly for the cephalometry rubric. Students generally reported positive perceptions and were positively associated with selected academic performance indicators, particularly in the cephalometry activity. Students generally reported positive perceptions regarding the use of rubrics as assessment tools.

These findings provide preliminary evidence supporting rubric-based assessment in preclinical orthodontic education. Future research should include multicenter studies, multiple evaluators to assess inter-rater reliability, longitudinal analyses of score stability, and psychometric methods specifically designed for ordinal data.

## Figures and Tables

**Table 1:** Changes in perception in the surveys on the wire bending rubric.

Items	Mean (SD), Pre	Mean (SD), Post	Post–Pre Difference	*t*-Test	*p*	*d*
**Total score**	**4.16 (0.61)**	**3.75 (0.77)**	**−0.40**	**5.47**	**<0.01**	**0.44**
**Theoretical knowledge**	3.52 (1.07)	2.92 (1.38)	−0.60	4.41	<0.01	0.35
**Manual skills**	4.70 (0.61)	4.33 (0.84)	−0.37	4.73	<0.01	0.38
**Objectives**	4.24 (0.82)	3.79 (1.01)	−0.45	4.24	<0.01	0.34
**Performance**	4.20 (0.81)	3.94 (0.95)	−0.26	2.95	<0.01	0.24
**Expectations**	4.13 (0.85)	3.78 (0.99)	−0.35	4.35	<0.01	0.35

**Table 2:** Changes in perception regarding the cephalometry rubric.

Items	Mean (SD), Pre	Mean (SD), Post	Post–Pre Difference	*t*-Test	*p*	*d*
**Total score**	**4.16 (0.58)**	**3.75 (0.60)**	**−0.07**	**1.3**	**0.30**	**-**
**Theoretical knowledge**	4.29 (0.83)	4.24 (0.74)	−0.05	0.63	0.53	-
**Manual skills**	3.87 (1.00)	3.87 (1.01)	0.00	0.00	1.00	-
**Objectives**	4.31 (0.71)	4.16 (0.83)	−0.15	1.91	0.06	0.15
**Performance**	4.17 (0.78)	3.98 (0.93)	−0.19	2.10	<0.05	0.17
**Expectations**	4.17 (0.75)	4.25 (0.80)	0.04	−0.51	0.61	-

**Table 3:** Cronbach’s alpha values.

Variable	Cronbach’s Alpha (α)
**Wire bending rubric**	**0.61**
**Cephalometry rubric**	**0.71**
**Pre-assessment** wire bending **survey**	0.77
**Post-assessment** wire bending **survey**	0.79
**Pre-assessment cephalometry survey**	0.76
**Post-assessment cephalometry survey**	0.73

**Table 4:** Item analysis for the wire bending rubric.

Item–Total Statistics			
**Scale mean when the item was deleted**	Scale variance when the item was deleted	Corrected item–total correlation	Cronbach’s alpha when the item was deleted
**5.57**	1.89	0.45	0.47
**5.27**	2.10	0.39	0.55
**5.38**	2.04	0.42	0.52

**Table 5:** Analysis of the functioning of items for the cephalometry rubric.

Item–Total Statistics				
	**Scale mean when the item was deleted**	Scale variance when the item was deleted	Corrected item–total correlation	Cronbach’s alpha when the element was deleted
**Tracing**	**11.16**	7.87	0.48	0.66
**Points**	**10.46**	7.07	0.65	0.58
**Lines and planes**	**10.71**	7.54	0.43	0.68
**Measurements**	**11.21**	7.62	0.43	0.68
**Cleanliness**	**10.76**	8.40	0.36	0.70

**Table 6:** Factor loadings for the wire-bending rubric.

Factor Loadings	
Design	0.78
Notches	0.72
Plane	0.75

**Table 7:** Factor loads of the cephalometry rubric.

Factor Loadings	
**Tracing**	0.71
**Points**	0.84
**Lines and planes**	0.64
**Measurements**	0.66
**Cleanliness**	0.55

## Data Availability

The raw data presented in this study are available on request from the corresponding author.
